# Participation in patient support forums may put rare disease patient data at risk of re-identification

**DOI:** 10.1186/s13023-020-01497-3

**Published:** 2020-08-31

**Authors:** James Gow, Colin Moffatt, Jamie Blackport

**Affiliations:** 1 Dartmouth College, Hanover, 03755 New Hampshire USA; 2 Faculty of Health and Life Sciences, de Montfort University, The Gateway, Leicester, UK; 3 Mirador Analytics Ltd. Priorwood House, Melrose, TD6 9EF UK

**Keywords:** HIPAA, Healthcare, Privacy, Rare disease, Support forums

## Abstract

**Background:**

Rare disease patients often struggle to find both medical advice and emotional support for their diagnosis. Consequently, many rare disease patient support forums have appeared on hospital webpages, social media sites, and on rare disease foundation sites. However, we argue that engagement in these groups may pose a healthcare data privacy threat to many participants, since it makes a series of patient indirect identifiers ‘readily available’ in combination with rare disease conditions. This information produces a risk of re-identification because it may allow a motivated attacker to use the unique combination of a patient’s identifiers and disease condition to re-identify them in anonymized data.

**Results:**

To assess this risk of re-identification, patient direct and indirect identifiers were mined from patient support forums for 80 patients across eight rare diseases. This data mining consisted of scanning patient testimonials, social media sites, and public records for the collection of identifiers linked to a rare disease patient. The number of people in the United States that may share each patient’s combination of marital status, 3-digit ZIP code, age, and sex, as well as their rare disease condition, was then estimated, as such information is commonly found in health records which have undergone de-identification by HIPAA’s ‘Safe Harbor.’ The study showed that by these estimations, nearly 75% of patients could be at high risk for re-identification in healthcare datasets in which they appear, due to their unique combination of identifiers.

**Conclusions:**

The results of this study show that these rare disease patients, due to their choice to provide support for their community, are putting all their healthcare data at risk of re-identification. This paper demonstrates how simple adjustments to participation guidelines in such support forums, in combination with improved privacy measures at the organizational level, could mitigate this risk of re-identification. Additionally, this paper suggests the potential for future investigation into consideration of certain ‘risky’ International Classification of Diseases (ICD) codes as quasi-identifiers in de-identified datasets to further protect patients’ privacy, while maintaining the utility of such rare disease support groups.

## Background

Most current literature on rare diseases highlights the need for improved data sharing among researchers and patients alike, as accessible data is critical for treatment development and serves as a platform to improve patients’ understanding of their condition [1–6]. As such, support groups and websites have been created for many of the over 7,000 ‘Rare and Orphan Diseases’ recognized by the United States (which defines Rare and Orphan Diseases as those with fewer than 200,000 cases in the United States in the Orphan Drug Act of 1983) [7]. As these groups continue to grow, patients post an increasing number of diagnosis/treatment stories in these open access forums to discuss treatment options and to provide hope and emotional support for other patients, all with the expectation that these “institutions will and should recognize their right for their privacy” [2, 8]. However, this expectation of privacy may be without foundation, as these patients’ data (both indirect and direct identifiers) may be extremely discoverable and, via their diagnosis/treatment stories, linked to their disease condition. A direct identifier, as it is used here, is defined as any piece of information that uniquely describes one individual, such as social security number or phone number. On the other hand, an indirect (quasi) identifier is a “feature that can indirectly identify individuals, such as their date of birth, death, clinic visit, residence postal code, and ethnicity,” when taken in combination with other indirect identifiers [3]. Thus, the ability to find a patient’s name and other direct identifiers in combination with a series of quasi-identifiers and their rare disease condition makes that patient potentially identifiable in de-identified healthcare data and, thus, may expose all their healthcare data beyond just their rare disease condition. This potential for re-identification is a product of the high likelihood of rare disease patients having combinations of quasi-identifiers that are unique in the US population as a consequence of the particularly low prevalences of their conditions. This uniqueness increases the chance of success for a motivated attacker attempting to re-identify records via prosecutor, marketer, or journalist attack and, therefore, increases the risk of re-identification [9].

The bad actor, or motivated attacker, in such attacks may either have gained access to the data via a data breach, the number of which is rapidly increasing in the modern era, or may actually be a member of the organization that is the intended recipient of the data [10]. However, whether access is obtained by breaching Health Insurance Portability and Accountability Act (HIPAA) Security Rule, which implements administrative, physical, and technical safeguards to protect access to electronically stored Protected Health Information (PHI), or by having the necessary permissions, this potential for re-identification should be prevented by HIPAA Privacy Rule. Enacted into law in 2002, Privacy Rule provides the requirements for anonymization of healthcare data by business associates and covered entities handling or producing de-identified data prior to its dissemination, with the aim of minimizing the potential for re-identification [11]. HIPAA Privacy Rule provides two avenues by which these entities can designate their datasets as sufficiently de-identified, Expert Determination and ‘Safe Harbor’ [12].

The ‘Safe Harbor’ method requires the data to have a series of 18 identifiers redacted completely; for example 5-digit ZIP codes, names, addresses, social security numbers, birth dates, etc [12]. Yet, ‘Safe Harbor’ may fail to limit the potential for re-identification of rare disease patients as a result of the absence of consideration of *rare disease condition* or *ICD-10 code*, the standard code set for diagnostics in the US healthcare setting, in its list of identifiers. More so, the second method, Expert Determination, is the verification that a dataset has a sufficiently small risk of re-identification by an expert with sufficient knowledge of statistics [12]. As with ‘Safe Harbor’, experts may not protect against the re-identification of rare disease patient records as a result of their potential exclusion of disease condition as a high-risk quasi-identifier. This is in accordance with Malin’s assertion that patient disease condition is only “accessible to a much smaller set of people” and, therefore, does not need to be considered ‘reasonably available’ [11].

Thus, this observational study first examined whether rare disease condition was, in contrast with Malin’s assertion, ‘reasonably available’ via rare disease support groups, and, how this distinction may affect the re-identification risk of rare disease patient health information. Further, it addresses the role this may play in the privacy risk of healthcare datasets on the whole, and whether this may impact the assessment of such large datasets.

## Results

**Fig. 1:**
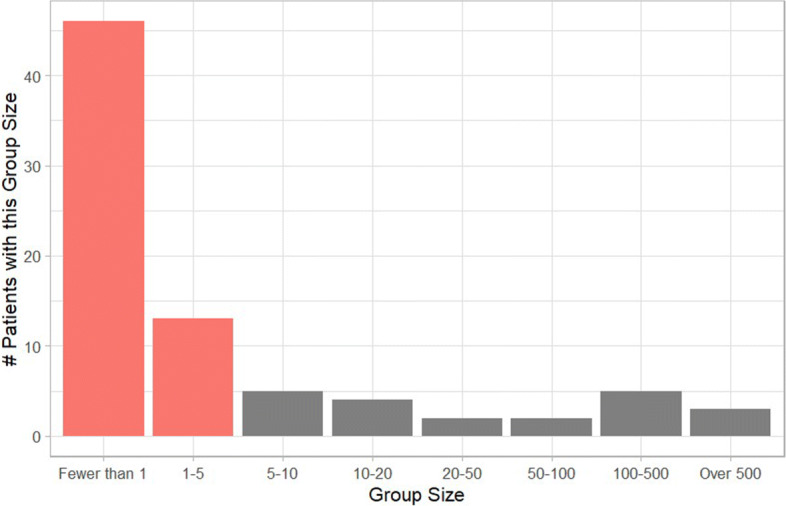
Group Size for Analyzed Patients. The x axis represents the number of people in the US population that share the patient’s combination of indirect identifiers. The y axis is the number of patients in this study that were a part of a group of that size. Highlighted in red are the two group sizes which constitute a high risk for re-identification

Initial qualitative assessment of the tabulated patient identifiers revealed that even more identifiers had been collected for most patients than was initially hypothesized. Figure 4, shown in the methods section, exemplifies the depth of information gathered for many patients but, in addition, quasi-identifiers like race, ethnicity, employment, physician, parent/children/sibling names, and even direct identifiers, such as social security number or phone number were gathered in some cases. It is challenging to accurately assess the impact of each of these variables on privacy risk; however, there is a more straightforward assessment possible for ZIP code, marital status, sex, and age, all of which frequently appear in anonymized datasets and have well documented reference data. Thus, following the protocol outlined in ‘Methods: Analysis Method,’ it was revealed that, based on those aforementioned identifiers and patient disease status, 73.75% of patient support group participants were in groups of less than five people and, therefore, are at great risk of re-identification. Looking at Fig. 1, the first two columns are group sizes of fewer than one and one to five, respectively (both shown in red), with the number of support group participants that had that group size on the y axis. A group size of less than one denotes that there are more identifiers present than is necessary to uniquely identify a single individual. This shows that not only are most patients at high-risk, but that 57.5% of patients were in a group size of less than one and hence are likely to be *the only* person with their group of indirect identifiers in the entire US population. This number may be slightly artificially inflated as the study did include some dead patients, but it is important to note that dead patients are inherently more discoverable as a result of the increased ability to collect data from obituaries and the increased redactions that usually accompany date of death in de-identified HIPAA-compliant datasets. However, even when removing the dead patients from this observational study, 69.6% of patients were still in high-risk groups of less than five people.

As for those patients who do not appear to be at a high risk of re-identification based on this preliminary analysis, only three were in groups of greater than 500 people. For these patient support group participants, it is likely that if an additional indirect identifier such as race/ethnicity and/or date of service were to be revealed by their participation, they too would be reduced to a group size under five and, therefore, be at great risk of re-identification. This is as a result of the fact that these additional indirect identifiers will often also appear in de-identified data and, thus, allows for a further subdivision of groups. In the case of race, this is quite a common variable seen in healthcare data, and, for date of service, although ‘Safe Harbor’ forbids exact dates, it is ambiguous whether less precise forms of date of service are allowed to remain. Furthermore, Expert Determinations may often also permit date of service to remain in de-identified data.

If race were to appear in a patient diagnosis/treatment story, the aforementioned age, sex, ZIP, marital status, and rare disease groups could be further divided into nine subgroups based on the nine race groups identified in the US Census.

This subdivision is even more pronounced for those patients with a service date (and potentially location) posted in their support group. Acknowledging that procedures may not occur every day of the year as a consequence of holidays, vacations, etc., we can conservatively estimate that there are at least 200 days on which any procedure may be performed in a given year, and each day is as likely as any other. This means that (ignoring the role of year and location) each patient group size would be divided by 200 for any patient who had a service date found during data collection, which was nearly every patient. As such, any patient support group participant in a group size of under 1000 that has this service date information, would now be at high risk of re-identification.

**Fig. 2:**
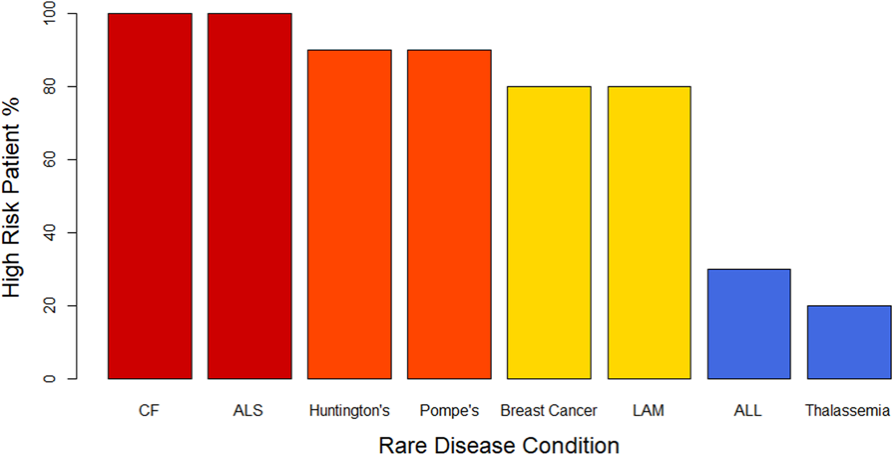
High-risk Patients by Disease Condition. The x axis represents the patient’s rare disease condition and the y axis represents what percentage of patients with the condition were in high-risk groups. For conditions with 100% high-risk patients the bars are coloured red, for 90% they are orange, for 80% they are yellow, and for <50% they are coloured blue

Further, it becomes apparent when looking at the data arranged by disease that there is a correlation between rare disease condition and patient group size, as shown in Fig. 2. This interdependency is primarily a result of the difference in disease prevalences. To exemplify this, when looking at the 20 patients in the study with the most prevalent rare diseases, only 25% of patients were at high risk (only about a third of the overall percentage of patients in high-risk groups). Additionally, there may be a secondary influence on this interdependency: the varying likelihood of patients to reveal certain indirect identifiers as a result of their disease condition. However, the sample size is too small in this study to substantiate this claim.

## Discussion

This preliminary investigation into the privacy risk of patient support group participation demonstrates that, under HIPAA’s assertion of ‘reasonably available’, rare disease condition should be considered a quasi-identifier for patients who participate in such forums, due to the discoverability of patients’ direct and indirect identifiers linked with their disease condition. Additionally, it provides an empirical basis for suggesting that patients should be better protected in their participation in such groups, so as to maintain their utility in the progression of the treatment of rare diseases and patient emotional support, but still maintain individual data privacy. Regardless of whether these patients comprise a large enough cohort to represent a considerable risk for large datasets as a whole, their healthcare data is linkable uniquely to them from de-identified datasets. This means that although a patient may consent to revealing their rare disease condition by posting a patient diagnosis/treatment story, their other health information may become known through their participation (i.e. James Roe may consent to revealing his battle with thalassemia, but through de-identification may unwittingly reveal his HIV as well).

This potential for re-identification can be reduced by a few simple changes to diagnosis stories/support-group posts at the patient level. Dates of service should always be avoided, especially beyond the granularity of year. Likewise, place of service and physician should not be posted in these publicly accessible settings. Perhaps most important is refraining from giving any place of residence more granular than state, and only identifying themselves by first name wherever possible. At an organizational level, be it hospital website or Facebook support group, the above recommendations should be provided as participation guidelines to all patients, and perhaps even enforced rules. Alternatively, these organizations could replace or obscure identifiers posting in their support forums. Additionally, it would be apt for Facebook groups and other membership-based support groups to make their pages private and provide at least a minimal screening process before admitting new members into the group. The value of this small change was made clear when, early on in the investigation, severe combined immunodeficiency (SCID) was considered as a potential condition for the study. Ultimately, SCID was not included in the study because the major Facebook support group was private and, thus, not viewable to nonmembers. As such, the acquisition of data on these patients was impossible from the Facebook source without requesting to join the group, pending the approval of the page administrators. This is a clear example of how the execution of a simple privacy mechanism can protect rare disease patients without compromising the utility of the patient support groups.

A further consideration is the differential number of available patient stories for each disease condition. Although not quantified, this study revealed that many more patient stories could be collected without excessive effort for some rare disease conditions like cystic fibrosis (CF), acute lymphocytic leukemia (ALL), Huntington’s chorea, and male breast cancer.

On the contrary, for Pompe’s disease and lymphangioleiomyomatosis (LAM) it was a significant time investment to find only the three to four sources (each with only a handful of stories) used in this study. This is significant as it indicates that for LAM and Pompe’s disease, although a high proportion of these patient stories are at high risk for re-identification in a healthcare dataset, their respective ICD codes of E74.02 and J84.81 are unlikely to be able to be used to re-identify many records in a de-identified dataset. On the other hand, some ICD-10 codes, such as those for CF and Huntington’s, can be linked to hundreds or thousands of patient stories and, thus, may warrant further consideration about their ability to re-identify health records.

However, as of yet, this study cannot suggest a method for the change in assessing the privacy risk of rare disease condition, but rather simply aims to better realize the existence of such risk.

**Fig. 3:**
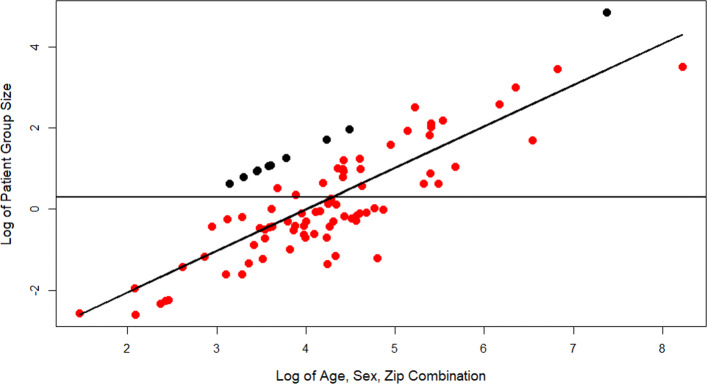
Scatter Plot of Patient Group Size and Age/Sex/ZIP Group Size. The x axis is the Log base 10 value of the number of people in the US that share each patient’s age, sex, and 3-digit ZIP as was collected. The y axis is the Log base 10 of their group size after incorporating the role of disease condition. Rare disease patients are shown in red and the control group of non-rare disease (multiple sclerosis) patients are in black. The horizontal line is at the value 0.301 which equals the Log base 10 of 5. The linear regression line with R-squared equal to 0.7695 for the rare disease patients is also shown

Indeed, very few ICD-10 codes are actually likely to add sufficient risk to datasets to necessitate further consideration as threats to the HIPAA compliance of data, as a condition must have both a sufficiently low prevalence and a high enough number of patient support stories in order to pose quantifiable risk. For instance, applying the analytic method to a low prevalence (one million US cases) - but not rare - disease called multiple sclerosis (MS) resulted in 10 patients, none of whom were at high risk. This is a consequence of a difference in prevalence rather than ability to collect data, as shown in Fig. 3. Likewise, although LAM had 80% high-risk patients in this study, if fewer than 100 LAM diagnosis/treatment stories are available, there is less than a 0.2% chance that a LAM patient in a claims dataset can be identified using their ICD code. However, despite the minimal application of the treatment of ICD-10 code as ‘reasonably available’ to compliance with the minimum standard of HIPAA, simple improvements in binning age, geographic locations, or even partially redacting high-risk ICD codes should be considered to better protect the privacy of those rare disease patients that have valorously posted their experiences in patient support forums.

**Fig. 4:**
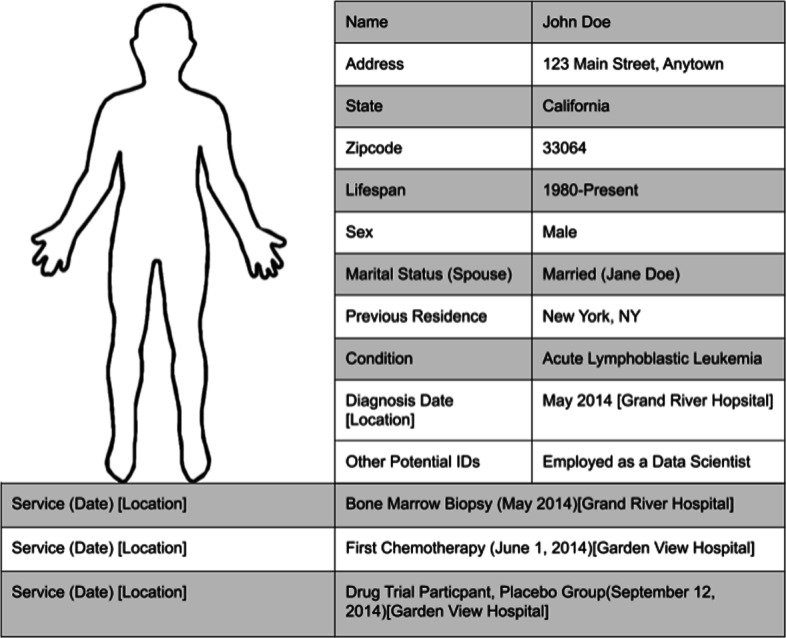
Tabulated Example of the Collected Patient Direct and Indirect Identifiers. The left column represents the direct or indirect identifier collected and the right column provides an example of how a structured record may appear for a sample patient

***Limitations of the Study***

When recording the data, certain assumptions of the validity of data had to be made. When using Facebook to answer queries on any given patient, the information displayed by the Facebook user was considered to be genuine and truthful (i.e. that their listed address was indeed their place of residence). However, most fields, especially place of residence, were confirmed by multiple sources during the investigation. Patient recollections were also assumed to be accurate, so data such as patient accounts of service dates or locations were not validated elsewhere. Finally, media sources were considered to be reputable sources of patient data, thus, any indirect or direct identifiers revealed by media sources were considered accurate.

Further, this study was performed by a manual data scraping technique which assumes that a presumptive attacker would be willing/able to invest significant time into data scraping prior to attempting a data breach. However, with the expansion of knowledge graphs and data mining tools, this ability to categorize unstructured data may rapidly accelerate the ability of attackers to breach healthcare data without a significant time commitment.

Lastly, this study did not attempt to re-identify rare disease patients in actual de-identified claims data, as this would have been a breach of privacy. Therefore, it assessed privacy based on variable fields (Age, Sex, ZIP, ICD-10 code) assumed to be present in most healthcare datasets, and was predicated on the assumption that rare disease patients will exist in healthcare datasets as a result of their frequent medical treatments.

## Conclusions

This study revealed that patients who participate in a rare disease support forum often reveal enough information about themselves such that a motivated attacker could be successful in re-identifying their data in healthcare datasets in which they appear. As such, participants may indeed be at a significant risk of having healthcare data beyond their rare disease condition (such as other diseases, disabilities, or procedures) revealed from de-identified data without their consent or knowledge. More so, this potential for patient re-identification produces adverse effects not just at the patient level, but also has the potential to significantly impact the disclosure risk of larger datasets including trial data, particularly datasets or trial data composed of only rare disease patients. This risk of participation in such forums can be mitigated by increased guidelines surrounding posting, as detailed in the Discussion, in tandem with increased privacy measures at the organizational level. As well as implementing a regulatory framework to prevent such risk of re-identification, organizations hosting patient support groups should also take steps to engage in educational efforts to inform rare disease communities as to the risk of the disclosing their indirect identifiers in tandem with their rare disease condition.

Indeed, it may also be prudent to restrict the indirect identifiers that appear alongside certain ‘risky’ ICD diagnosis codes, which are those that code for a rare disease with low prevalence but a high number of rare disease support group participants. Alternatively, these risky ICD-10-CM codes could be binned or grouped such that the prevalence of the combined group of ICD codes was sufficient such that they would no longer uniquely identify individual patients. This binning could proceed by grouping multiple risky ICD codes or grouping a risky ICD code with a non-risky ICD code for a rare disease with similar characteristics. Such restrictions could maximize the utility of the data for rare disease research by still retaining a description of rare disease condition and its related effects while also protecting individual’s anonymity, although a larger study would be required to assess the need for such a measure. In summary, rare disease support groups provide an essential function to disseminate information and provide emotional support and, therefore, it is important to maintain these groups’ utility while protecting participants from incurring an increased risk of re-identification to their healthcare data. Thus, the measures described above should be implemented to protect these patients’ data while allowing for continued use of such groups to provide much needed support in rare disease communities.

## Methods

The disease condition and publicly available associated direct and indirect identifiers of 80 patients with eight different rare diseases were collected via simple internet queries beginning with a patient support group post or a diagnosis/treatment story. Each combination of patient indirect identifiers was then assessed for its risk of re-identification using a method of privacy assessment that assesses each patient in the context of the entire US population. This was performed in place of the widely accepted method of k-anonymity in order to eliminate sample size bias from the study [13]. Prior to starting data collection, the following structure for the data scraping process was defined to minimize investigator bias and, thus, increase the ability of the collected sample to represent the total population of patient support group participants.

### Data Collection Method

#### Selecting the Disease Conditions

Diseases were selected as those with high public awareness (due to a significant social media, media, or pop culture presence) and/or a prominent place in the rare disease research community. This was done in order to assess those diseases for which there is the most publicly available patient data and, thus, the privacy risk is the most substantial. This led to the selection of the following conditions:

1. Male breast cancer: selected for the numerous support groups and large social media presence of the disease
2. Amyotrophic lateral sclerosis (ALS): developed infamy in America due to Lou Gehrig and the ALS ‘Ice Bucket Challenge’
3. Pompe’s disease: well known due to the movie *Extended Measure* and fund-raising campaign/research efforts of biotech CEO John Crowley
4. Thalassemia: well known due the variety of professional athletes with sickle cell trait promoting the cause
5. Lymphangioleiomyomatosis (LAM): growing notoriety due to the research effort from University of Pennsylvania and appearance in pop culture TV show *House*
6. Acute lymphocytic leukemia (ALL): high levels of media attention as a result of its prevalence in young children
7. Cystic fibrosis (CF): its devastating congenital effects made it a research focal point, including that of the 2007 Nobel Laureates in Medicine, which led to its appearance in pop culture including the 2019 film *Five Feet Apart*
8. Huntington’s chorea (HD): appeared across pop culture due to its high mortality and the rapid deterioration of famous folk singer Woody Guthrie. Examples include *Breaking Bad*, *ER*, and *Scrubs* as well as books like *Double Helix*

#### Defining the Search Terms

To optimize the data collection process, the search terms were defined for each condition prior to data collection. Two sources of patients were used. The first was patient story collections from an initial Google search of “ <Disease Condition > Patient Stories.” The second was Facebook, used to examine the role of social media in patient support. Pages/Groups were found by entering “ <Disease Condition > Support Group” into the search bar of Facebook in order to identify the most popular group or page related to the rare disease. Facebook Groups are private or public Facebook forums where all members can post, comment, and view other members in the group and are managed by the Facebook user who founded the group, and any additional administrators. Facebook pages are open forums where only the administrator can post but any user can like the page to view its updates and can comment and interact with its posts.

#### Establishing the Fields for Data Collection

Based on a preliminary review of key indirect identifiers that may make a rare disease patient uniquely identifiable in an anonymized dataset, it was determined that the direct and indirect identifiers shown in Fig. 4 would be collected, where present, for each patient with a rare disease condition used in the study [11].

Figure 4 exemplifies what the outcome of such a collection may be for a representative patient in the second column. If the patient’s exact 5-digit ZIP code was not found, their 3-digit ZIP (or multiple 3-digit ZIPs if need) was recorded. Some patients in the study were deceased, in this case the date of death was recorded. The Other Potential IDs category is a conglomerate of less frequently appearing quasi-identifiers such as employment and educational background as well as direct identifiers such as phone number and social security number.

#### Collecting Data: Selecting Patients & Manual Data Scraping

For each disease, patients from both Facebook support groups and websites with collections of patient diagnosis/treatment stories were included in the study. The process of selection differed between these two platforms but, in both cases, the process of patient selection was as random as possible to limit selection bias.

In order to locate collections of patient accounts of diagnosis/treatment processes, links were chosen indiscriminately from the first 10 pages of Google search results from the aforementioned search. This process was used in favor of utilizing the resulting links in order of appearance as the patient stories appearing earlier in Google searches may contain a bias towards increased patient discoverability. The links to patient story collections included hospital websites, disease foundation sites, fundraising pages, and media articles. Within websites that contained multiple patient stories, three or fewer patients were selected unsystematically from the displayed list. For example, if a link to the Garden View Hospital Cystic Fibrosis Diagnosis Stories link was accessed from page seven of the Google search for “Cystic Fibrosis Patient Stories”, and it contained 15 listed patient stories, the third, tenth, and eleventh story might be used.

Concomitantly, a Facebook support group for each disease was accessed and posts under the Community, Posts, or Discussion links were investigated. Facebook orders posts chronologically which does not introduce a bias to increased discoverability, thus, there was no need to scramble the order in which posts were investigated. When a post was encountered that was greater than 200 characters in length and was regarding a patient’s diagnosis or treatment with the disease of interest, that person was included in the study. However, posts about children, other family members, or friends were not included in the study. Comments on original posts selected for containing a potentially discoverable patient were read for further information on the patient of interest, but not for other potentially discoverable patients. As with each collection of patient stories above, no more than three patients were found on any one Facebook group.

After a patient was selected, their name was recorded along with the information that the original post or article contained pertaining to the fields aforementioned. Following (in no particular order), internet queries were made as to birth and death records as well as marriage certificates for each individual using web-based search engines and on-line local obituaries. An attempt to locate the patient’s profile on Facebook was then made either by clicking a profile hyperlink in a patient support Facebook group if present, doing a city specific name search, or searching posts containing the patient’s name and their disease condition. If a profile was found, the “About” section was used to collect further identifiers and the patient’s public posts were quickly scanned for other relevant information. According to Facebook’s Data Policy, it is each user’s responsibility to regulate the viewing permission of the information and content users provide. Hence, they clearly state that public information in a profile, Facebook page, Marketplace, or Facebook group can be viewed and collected by anyone and, therefore, this data is openly available to any potential attacker of healthcare data, even if they are not a Facebook user.

Continuing, if address or town of residence could be determined along with age from the original source and/or further investigation, White Pages was used to search for the patient. White Pages is a web-based search engine that compiles multiple public record sources including, but not limited to, telephone records, public utilities, voter’s registration, and state licensing agencies. If there was a full name match within the patient’s known location and of the appropriate age on White Pages, then the profile was considered a potential match. If a potential match had at least one further confirmation of identify to ensure it was a match for the patient in question, it was then considered a matching record. Identity confirmations include a listed alias (i.e. a woman’s maiden name), any known siblings/parents/children listed as related individuals, a previous address that matches the patient’s known hometown, a previous location of residence, or a listed spouse. If the identity was confirmed to match the patient, any additional information not already linked to the patient was recorded. A brief Google search of the patient’s name combined with their disease name was also used to reveal any other media or social media sources that may contain pertinent information and potentially an obituary for those few patients that had already passed away.

#### Example of Data Collection for a Selected Patient

To provide clarity on how indirect identifiers were gathered, the following is the description of data collection for a sample patie nt diagnosed with thalassemia, who we will refer to as James Roe. All real information has been replaced by artificial data so as to describe the process without compromising the patient’s identity and data.

“Thalassemia patient stories” was entered into the Google search bar and a link to a Thalassemia Support Association webpage was selected unsystematically from the first page of the Google results. The link *James’s Diagnosis Story* was arbitrarily chosen from the list. James’s story had a head-shot photo at the top, and was an account of his initial diagnosis and treatment. The story revealed he had received his diagnosis in the 4th grade and that he had now been living with thalassemia for 20 years. The story also revealed that he currently lived in California and discussed his initial visits to Garden View Hospital as a child to receive blood transfusions. Although this was an unusually vague story as compared with many other patients’ accounts of their diagnosis, it provided the basis for gathering much more information.

First, the date of the story’s publication was available; thus, knowing James was eight or nine at the time of diagnosis (and that it had been 20 years since) his approximate age could be determined. Additionally, at the end of the story, James wrote briefly about his participation in a 5k race to raise money for thalassemia. Using this information, James’s old “GoFundMe” fundraising page for this 5k race could be found, which had his name, location, and the same picture as his original diagnosis story. This page had a link to another diagnosis story James had posted, but this page had since been taken down. However, the fund-raiser page also revealed that James’s last name was Roe. Now entering James Roe, thalassemia into LinkedIn (a social network focused on professional networking) produced only a couple of results. The top result for this query was a profile that matched James Roe’s full name, had a picture of James as the profile photo, listed California as his place of residence, had a listed affiliation with the same thalassmeia society on whose page James’s initial story was posted, and had written in the bio that he has been diagnosed with thalassemia; a clear match for the patient James Roe. LinkedIn also provided information on James’s employment status as a sales associate at a large firm, along with a link to his work profile, which revealed James lived in Anytown, California and used to live on Main Street in San Francisco.

It was trivial to find James on Facebook, but his “About” section was not public. However, on his page, James had marriage photos with unrestricted viewing settings (i.e. viewable to any Facebook user), which obviously revealed his marital status and allowed for the discovery of the Facebook page details of his spouse, Mary Roe. Her Facebook page confirmed James’s marriage date and marital status as *married*. James’s Facebook page also revealed he was originally from West Lebanon, New Hampshire. Now knowing James’s approximate age, full name, and three towns of residence, it was possible to use White Pages. Entering James Roe Anytown, California into White Pages produced only one full name and town match. This profile also matched James’s age. Under “Relations” it also listed Mary Roe, his wife, and some family members that were listed on his Facebook. The White Pages profile was, thus, clearly a match, and so could be used to obtain James’s exact street address, 5-digit ZIP code, and telephone number.

## Analysis Method

***Patient’s ZIP Code, Sex, and Age Group as Indirect Identifiers***

Each combination of quasi-identifiers was analyzed to determine whether a patient’s collection of indirect identifiers in combination with their rare disease condition is likely to be unique in the US population (i.e. is the patient likely to be the only 36-year-old male in the ZIP 017 with cystic fibrosis). If the patient’s combination of quasi-identifiers is unique, or in a small group of less than five individuals, then de-identified data containing their data row with these identifiers would be considered high risk for re-identification. This threshold group size of five comes from the frequent use of five-anonymity in Expert Determinations when using k-anonymity to assess re-identification risk of anonymized datasets [14, 15]. This method was used to assess the privacy risk of the data in place of k-anonymity to mitigate the influence of sample size, which has been a challenge previous studies have faced in risk assessments done on rare disease data [16]. This more conservative risk assessment allows for the extrapolation of the conclusions made in this study to any real healthcare datasets in which rare disease patients may appear.

In order to compare each record to all other potential records of these indirect identifiers that exist in the US population, reference data was compiled concerning the number of people of each age and sex that live in each 3-digit ZIP. This was done by using the American census data FactFinder to generate tables of population within each 5-digit ZIP code sorted by five-year age grouping (with age being capped at 85) and sex [17]. The 5-digit ZIP code populations were summed across each 3-digit ZIP, producing the initial reference table, because, although many patient 5-digit ZIP codes had been found during data collection, most HIPAA-compliant, de-identified data has ZIP redacted to three digits [3, 11, 12, 15]. These census tables were produced based on estimated values for the 2017 populations which in turn were based on the growth rate being applied to the 2010 census. It so happened that the collected data did not contain any patients in small 3-digit ZIP codes (population <20,000) which would have been redacted by ’Safe Harbor’ data and, therefore, each patient’s actual 3-digit ZIP (or group of potential 3-digit ZIPs) was used. If no ZIP code was recorded, but a state of residence was known, then the United States Postal Service website describing the American 3-digit ZIP codes was used to find all potential 3-digit ZIP codes for that state and the populations matching the appropriate age group and sex of the patient were summed across all those possibilities [18]. To match each patient’s age to the reference data, the patient ages were calculated for what it would have been at the start of 2017, based on their birthday date/year.

For the few patients in the study that were dead in 2017, the aforementioned reference data could not be used as the census data is based on the living population and, therefore, dead patients would not be included. As such, dead patients were rather assessed as a part of a different set of reference data made up of only dead people. This reference data was the Center for Disease Control and Prevention (CDC) data on yearly deaths in each five-year age group by five-years at death divided by sex and county of residence at death (county is a geographic gradation slightly larger than 3-digit ZIP code) [19]. In the one case in which the patient death year was outside the range of CDC provided data (1999-2018), the summative value of all the deaths during that 20-year range was used as a conservative estimate.

***Incorporating Marital Status as a Quasi-Identifier***

After assessing each patient’s group size using the above reference data, marital status was incorporated as another potential indirect identifier. Marital status for this study was defined as ‘Married’ or ‘Not Married’, with the latter category encompassing separated, widowed, divorced, and never married individuals, and the former only those with a confirmed spouse. Again, the American census, was used to determine the reference values used for this study. This reference data on the number of Married and Not Married individuals in the US by age, sex, and ZIP allowed for the calculation of the probability that an individual was married with respect to the dependent variables of age, sex, and ZIP. In order to take the most conservative approach possible, so as to minimize Type I error, the estimates stated by the census FactFinder were summed with the provided margins of error calculated based on a 90 percent confidence interval to find the value of the upper bound. This is a more conservative approach as it produces an overestimate of both the marriage rate and ‘Not Married’ rate. For instance, the upper bound may estimate that in a ZIP code with 4,000 men age 50-55 there are 2,500 married men and 2,500 unmarried men. The marriage rate and ‘unmarried rate’ may both thus equal 62.5%. Therefore, it would consider each patient in the study to be in a larger group than is actually the CDC’s best estimate, which provides a more conservative estimate of group size.

In some cases, the marital status data table differed from the total population census data in its age groupings. The marital data separates the 15-19 age group into 15-17 and 18&19 and concatenates the 65-69 group with the 70-74 group and likewise with 75-79 and 80-84 groups. In these cases, prior to calculating the proportion that were married, the component groups were combined, using the root mean square sum rule to determine the appropriate Margin of Error to be used for the sum. This study assumed there were no married individuals under 15 (as does the census) and, thus, for patients younger than 15 (and any patient whose marital status was unknown) the original value generated for Age/Sex/ZIP was left unchanged. If the patient’s known age spanned more than one age group, the more conservative value was used. For example, if a patient was known to be 29-31 and married in February of 2017, and the census data showed a marriage rate of 30% for those aged 25-29 and 50% for those aged 30-34, then the value of 50% would be used. This is, as previously described, a deliberate attempt to minimize Type I error, despite its introduction of additional Type II error, so that any conclusions regarding a high risk of re-identification can be made confidently. If a patient’s marital status and ZIP were known, but the patient’s age was unknown, then the most conservative value for that ZIP code was used. If the patient was deceased by 2017, even if their marital status was known at the time of death, no proportion was applied and a multiplier of one was used.

***Incorporating Disease Condition as a Quasi-Identifier***

Each patient now had an associated group size, none of which were below the high-risk threshold of a group size of five. However, because their rare disease condition was made ‘reasonably available’ by their diagnosis/treatment stories, their disease condition must also be considered a quasi-identifier. Consequently, the prevalence of each patient’s respective disease condition was applied to address the effect of this quasi-identifier on their group size. However, there are no clearly established prevalences that could be used for these rare diseases due to the obstacles that exist to sufficient data collection in rare disease communities. As such, prevalences were calculated based on published incidences taking into account age and sex dependence where relevant.

First, since thalassemia is a congenital condition its prevalence is not age dependent, and its low mortality rate means it does not usually impact death rate in Americans. As such, its approximate incidence rate of 44/100,000 was simply applied as the prevalence. As with thalassemia, Pompe’s disease is congenital. However, Pompe patients do have a reduced life expectancy. Due to the rapid changes to this life expectancy over the last decade, it is impossible to approximate the difference in prevalence and incidence rate and, thus, in order to remain as conservative as possible, the incidence rate of 2.5/100,000 was used. For LAM, both the incidence and prevalence are extremely hard to estimate but prior studies have suggested its prevalence for women is in the range of 1/200,000 so this value was used across all age groups (only women were included for LAM in this study), although this is likely a gross overestimate of the prevalence for the younger age groups [20]. For cystic fibrosis, the 2017 Cystic Fibrosis Patient Registry was used to find the exact number of recorded CF cases in the US for the year 2017, which was 29,460 [21]. The prevalence used was, therefore, slightly more than 9/100,000 which was calculated using 327,200,000 as the estimate for the US population in 2017 [17]. This prevalence was used for both sexes and all ages as CF is congenital and has an autosomal inheritance pattern. For Huntington’s it is commonly reported that there were about 16,900 cases in the US in 2017. However, a recent epidemiological study on the disease has shown the prevalence to be nearly 9/100,000 when ignoring the fact of race [22]. As such, this study assumed there were 30,000 cases of Huntington’s in America to remain conservative. For ALS, epidemiological studies have an incidence rate of approximately 1.5/100,000, but there are no specific figures regarding prevalence [23]. Despite this, the ALS Association reported approximately 16,000 cases in the United States in 2017, which is a logical estimate given the aforementioned incidence rate and the approximate mortality rate of ALS [24]. Therefore, for ALS patients who were alive in 2017, a prevalence of 4.92/100,000 was used. Due to the high mortality rate of ALS, however, this is not an accurate estimation for those patients already deceased by 2017. Rather, for those patients that were deceased, a prevalence of 2/100,000 was used. This value was used because 60 people die annually of ALS in the United States, and about three million people die in the US each year in total [25]. As such, a person who died in any given year has a 60/3,000,000 chance of having died due to ALS [24].

For the final two conditions, male breast cancer and ALL, the estimation of prevalence was a more involved task as the prevalence varies greatly by age group due to age varying incidence and death rates for each age group. For male breast cancer, a 2018 study was used to estimate an incidence rate for each five-year age group [26]. Male populations in the US by age group were then estimated using Statista, as was the average death rate for each group [27]. Using these values, an approximate number of new diagnoses for each age group could be determined. Then, to estimate how many of these patients would continue to contribute to the patient population as they aged, the 5- and 10-year survival rates were applied as reported. It was then assumed, for the purposes of this study, that after 10 years of remission there was no significant increase in death rate as compared to the general populous. As such, the number of patients that made it from the 10-year survival to 15-year survival point (and 15-20, 20-25, etc) was assumed to decrease with age along the normal rate of death for each age group. This is a significant overestimation of the prevalence values, because it assumes that the current five/ten year survival rates (and modern whole population death rates) were the same up to 60 years ago, which is known to be false. However, these current rates were used so as to be as conservative as possible in the estimation of prevalences. If any patient spanned multiple age groups, then the more conservative estimate of prevalence was used. A similar approach was used for ALL, producing the same age specific prevalences, also incorporating the role of sex [28]. The ALL survival rates used were found from the St. Jude Hospital page and cancer.net; however, the values listed were independent of age at diagnosis [29, 30]. Additionally, although after 10 years of remission ALL patients are considered to be ‘cured’, they will be still be considered to be a part of the patient population until their death as the condition remains a part of their medical record.

## Data Availability

The data that supports the findings of this study is available from Mirador Analytics Ltd. but restrictions apply to the availability of this data due to its ability to potentially re-identify healthcare data of the patients in the study, and so it is not publicly available. Data is, however, available from the authors upon reasonable request and with permission of Mirador Analytics Ltd.
